# Need for cognition moderates the impairment of decision making caused by nightshift work in nurses

**DOI:** 10.1038/s41598-022-05843-2

**Published:** 2022-02-02

**Authors:** Jiaxi Peng, Huijie Lu, Jiaxi Zhang, Yongcong Shao, Lei Wang, Jing Lv

**Affiliations:** 1 School of Psychology, Beijing Sport University, Beijing, China; 2 Department of Military Medical Psychology, Air Force Medical University, Xi’an, China; 3 Xi’an Research Institute of High-Technology, Xi’an, China; 4 Department of Medical Psychology, Strategic Support Force Medical Center, Beijing, China; 5 Department of Psychology, The Second Medical Center and National Clinical Research Center for Geriatric Diseases, Chinese PLA General Hospital, Beijing, China

**Keywords:** Psychology, Human behaviour

## Abstract

The current study explores the effect of nightshift work on the decision-making competence and performance of the Iowa Gambling Task (IGT) and analyzes whether individual differences in the need for cognition (NFC) can moderate this effect. A total of 107 female nurses were recruited to complete the decision-making competence scale and IGT at two times, after a night shift and after a day shift. The results revealed that the IGT scores and decision-making competence of nurses after nightshift work significantly declined, and also that the decrease in decision-making competence was related to the nurses’ performance of the IGT. Additionally, the decreasing degree of IGT and decision-making competence scores of the high-NFC group were significantly lower than those of the low-NFC group after nightshift work. In can be concluded that the decrease in decision-making competence which was related with poor decision-making due to nightshift work. NFC moderated the effect of nightshift work on decision-making.

## Introduction

Nightshift work leads to mental fatigue^1^. Previous studies have documented that mental fatigue decreased alertness, learning, memory, and executing functions, and also induces anxiety, anger, boredom, and other negative emotions^2,3^. However, nightshift work is a normal operating condition commonly experienced by people in certain occupations, such as long-distance drivers, air controllers, and medical staff^4^.

### The effect of mental fatigue on decision-making

Decision-making is an advanced cognitive activity, and the effects of mental fatigue on decision-making have been extensively studied^5–7^. For instance, Castro and de Almondes found that physicians on the day shift schedule have good sleep quality and physicians on the nightshift schedule have bad sleep quality, and good sleep quality was related to a better performance in the Iowa Gambling Task (IGT). Sleep deprivation is the laboratory model for mental fatigue, and Killgore et al.^47^ found that after 49 h of sleep deprivation, participants performed worse in the IGT than they had previously, and they tended to make irrational decisions. Caffeine, dextroamphetamine, and modafinil have all been tested for their effectiveness in reducing the effect of sleep deprivation on alertness, but studies have documented that the intake of caffeine or other awakening accelerants did not efficiently improve the IGT scores of people in a state of sleep deprivation^8,9^.

Though previous research has implied that nightshift work or sleep deprivation will induce impaired decision-making in the IGT, the underlying mechanism has not been sufficiently explored. It remains unclear which cognition factors are associated with the irrational decision-making that people exhibit when in a state of fatigue. We also do not yet understand whether personality and other traits can moderate the effect of mental fatigue on decision-making.

### Decision-making competence

Decision-making competence (DMC) refers to the ability to make better decisions, as defined by the decision-making principles posited by models of “rational choice”^10,11^. Parker and Fischhoff^10^ summarized four core decision-making skills: (1) assessing beliefs: this skill determines whether decision-makers can effectively perceive the probability that an event will occur; (2) assessing values: this skill allows decision-makers to assess the consequences of options in a manner that is sensitive to values, but not to irrelevant information; (3) integration: this ability concerns whether or not decision-makers can accurately and rapidly master decision-making rules; rational decision-makers should quickly discover the decision-making rules and ratiocinate and judge according to the specified rules, rather than making decisions according to their personal preferences; and (4) metacognition: this skill involves whether decision-makers know if they have enough knowledge for the current task; they should not be blindly overconfident or underconfident. On this basis, Bruine de Bruin et al.^11^ compiled the Adult Decision Making Competence scale (A-DMC) and used six subscales to represent the above four core decision-making skills. Many studies have confirmed the validity of A-DMC for DMC assessment among adults^12,13^. Bruine de Bruin et al.^11^ found that individuals with lower A-DMC scores had lower social status, lower economic incomes, and lower education levels, and these individuals also demonstrated a higher rate of destructive behaviors, such as excessive drinking and drug abuse. In a longitudinal study by Parker et al.^12^, participants with low scores in terms of DMC measurement 11 years ago were found to be more likely to commit crimes, use drugs, smoke, and exhibit insecurity.

So far, there has been no study about sleep deficiency and DMC, but some studies have implied that irrational risk seeking after being in a state of mental fatigue may be related to the reduction of core decision-making skills^14^. For example, Venkatraman et al.^15^ found that the tendency to take risks after sleep deprivation was significantly related to enhanced ventromedial prefrontal cortex activity and to a decrease in anterior insula activation that occurred during the brain’s experience of reward outcomes. Namely, the risk tendency of individuals after sleep deprivation was associated with the alteration of feedback processing or assessment, indicating that the Assessing Values ability was lowered after sleep deprivation^15^. Nofsinger and Shank^14^ documented that individuals who get better sleep display less distortion of probability and have a lower discounting rate, suggesting that lack of sleep may influence belief assessment. Moreover, the same studies have explored the variations in decision-making confidence that may occur after sleep deprivation, but the findings are not consistent^16^. For instance, Fraser et al.^18^ found that people that were sleep deprived were marginally less confident as compared with those with a normal sleeping status. However, Baranski^16^ study found that the indices of confidence (i.e., calibration, resolution, over- and underconfidence), as well as the accuracy of the pre- and post-task estimates of performance, remained stable over the sleep deprivation (SD) period. The lack of significant change in confidence in Baranski’s study was probably that the task in the experiment had more time to think and participants had no time pressure and not be encouraged to respond faster. However, researcher support that participants in spite of having the ability to rally and response to some faster higher-risk stimuli, but there remains evidence of impairment and insensitivity to stimulation, regardless of the participants realize it or not^17,18^.

Thus, we propose hypothesis 1: *Nightshift work can lead to a reduction in core decision-making skills; namely, the decline of DMC.*

Parker and Fischhoff^10^ believed that a higher DMC led to more reasonable decision-making. Bad performance in the IGT is regarded as poor or impaired decision-making capacity in many studies^19,20^. Additionally, IGT performance is related to several cognitive components, such as feedback learning, probability estimation, emotion regulation, and working memory^21–23^, and some of these cognitive components are also assessed in terms of DMC. For example, probability estimation is analogous with assessing beliefs. Peng et al.^13^ found that the individuals with higher A-DMC scores performed better in the IGT.

Thus, we propose hypothesis 2: *IGT and DMC scores are positively associated, and lower scores in the IGT after nightshift work are related to a decrease in DMC.*

### Need for cognition

NFC is a trait variable that describes the degree to which people desire to engage in cognitively challenging tasks and effortful thinking^24,25^. Compared with low-NFC individuals, those with high NFC will spontaneously seek, collect, and analyze information and will devote more cognition resources and efforts to process information; in other words, high-NFC people have higher motivation to use cognitive resources^26^.

The effects of NFC on decision-making have been extensively studied^27,28^. Carnevale et al.^27^ found that NFC was significantly related to two subscales of DMC, including framing and honoring sunk costs. Harman^28^ reported that low NFC participants performed significantly worse in their decision-making than high NFC participants, because low NFC participants placed more importance on gains as opposed to losses when performing the IGT. Lack of sleep will lead to a decrease in cognitive resources^29^. NFC reflects the degree to which an individual is willing to use cognition resources to handle a problem. Thus, we can speculate that those with high NFC levels will invest more energy and effort into processing information, and those with low NFC levels put less effort into making decisions. Even with a deficit of cognition resources, high-NFC individuals were still more willing to actively use cognition resources for decision-making and thus were able to make more rational judgments, and those with low NFC were less willing to use cognition resources to solve problems and ultimately made judgments according to their emotions and intuition. Additionally, high-NFC individuals may be able to recruit additional resources for maintaining cognitive performance in a state of sleep deficiency^30^.

Therefore, we present hypothesis 3: *NFC may moderate the effect of nightshift work on DMC and IGT performance.*

### The aim of the current study

Based on previous studies, we aimed to discuss whether the decrease in IGT scores due to nightshift work is related to DMC decline, and to explore whether NFC can moderate the effect of nightshift work on decision-making. The nurses who staff in-patient hospital departments, because of the special needs that this occupation requires, must work night shifts and thus often suffer from long-term lack of sleep^31^. In the general hospitals of China, the nurses that staff the in-patient departments usually work a three-shift system: the day shift from 8:00 to 17:00, the swing shift from 17:00 to 23:00, and the night shift from 23:00 to 8:00 the next day. The nurses take the day shift, swing shift, and night shift alternately, and they work 5 days and rest 2 days every week. Nurses are not allowed to sleep or nap during their shifts, and they must patrol patients’ rooms on a specific schedule. As reported, nurses after the night shift feel more tired than they do after the day shift; they display more negative emotions, and their attention and memory is more impaired^32,33^. Hence, this study targets the nurses that staff hospitals’ in-patient departments. With a “self before-after contrast” design, we compare the IGT and DMC scores among nurses after the night shift and after normal resting. Participants are divided into a high-NFC group and a low-NFC group, and the effects of sleep deficiency on the IGT and DMC scores of the two groups are analyzed.

## Methods

### Participants and procedure

The participants include 107 female nurses of the inpatient departments of a large general hospital in Beijing, China. Their ages range from 23 to 34 years, with a mean of 25.33 years (SD = 2.17). At the time that we gathered the data, the participating nurses had been working in hospitals anywhere from 16 to 148 months, with a mean working period of 72.47 months (SD = 12.68). The participants were gathered and asked to fill in the NFC scale and demographic information.

To avoid the practice effect, we separated the participants randomly into two groups. In group A, the nurses worked the night shift from 23:00 to 8:00 the next day and finished a fatigue assessment, the IGT, and the A-DMC at 8:00. After 2 weeks, they worked the day shift (namely, they had rested normally the night before), and they began to fill out the IGT and DMC scales at 8:00. In group B, the participants first finished the IGT and DMC measurements during the day shift, and then they finished these measurements after the night shift 2 weeks later. All of participants were commanded to restrain themselves from taking any kind of stimulant drugs, caffeinated foods and beverages for 4 h prior to and during their shift. To ensure consistency between groups, the demographic differences between the two groups considered balance as far as possible when selected the subjects.

The participants completed the scales in quiet nurses’ rest rooms. They are all volunteers, and were rewarded with 300 RMB (about 45 USD) for their participation. The research described in this paper meets the ethical guidelines of the Beijing Sport University and has been approved by its ethics committee. All of the participants had read and signed the informed consent form before participating in the study.

## Measurements

### Stanford sleepiness scale (SSS)

The SSS consists of one question in which participants rate their degree of sleepiness on a scale ranging from 1 (not sleepy) to 7 (very sleepy)^34^.

### Iowa gambling task (IGT)

The IGT was designed by Bechara et al. with the purpose to explore how people make decisions under complicated or unclear conditions^35,36^. Participants viewed a computer monitor on which four decks of cards (labeled as A–D) were placed. In each trial, participants were asked to choose one card from one of the four decks, and a computer would give the feedback of a win or a loss. There were 100 such trials in total before the task was terminated, conducted in five trial blocks (each block consisted of 20 trials). Decks C and D were conservative and advantageous in the sense that rewards (i.e., a 50% chance of earning 50 points in Deck C and a 90% chance of earning 50 points in Deck D) and penalties (i.e., a 50% chance of losing 25–75 points in Deck C and a 10% chance of losing 250 points in Deck D) were small, tending to result in overall gain. In contrast, Decks A and B were risky and disadvantageous in the sense that rewards (i.e., a 50% chance of earning 100 points in Deck A and a 90% chance of gaining 100 points in Deck B) and penalties (a 50% chance of losing 35–150 points in Deck C and a 10% chance of losing 1250 points in Deck B) were large, leading to an overall loss. Performance on the IGT was assessed by the mean number of advantageous card selections minus the number of disadvantageous selections [(C + D)-(A + B)], both in each trial block and overall.

### Adult-decision-making competence scale (A-DMC)

For this study, the A-DMC developed by Bruine de Bruin et al.^11^ was translated into Chinese and tested for its reliability and validity^13^. The A-DMC consists of six sub-scales; namely, resistance to framing (RF), resistance to sunk costs (SC), consistency in risk perception (RP), recognition of social norms (SN), application of decision rules (DR), and under/overconfidence (UOC). In the RF sub-scale, participants are asked to rate preferences for 7 pairs of questions that are even in value but described in loss or gain frame differently. Scores are obtained by the mean absolute difference for the two problems. SC subscale (10 items) assess the ability of participants to ignore past disbursements when considering future events by asking them to select between an option that indicates the normatively correct choice versus one that regards sunk costs. Scores are obtained by the average assessing across items. RP subscale (20 items) is assessed as the consistency of risk estimates across time frames. SN subscale (16 items) involves a two-pronged task that first asks individuals to answer whether they would accept a negative event (such as stealing), and then asks participants to judge what percentage of people would accept that negative event. The scores are obtained by calculated the correlation between the probability that a participant accepts a negative event and the probability that people generally accept it. The DR sub-scale (15 items) requires the participants to choose different commodities according to the decision rule. One point is scored if the choice conforms to the decision rule; otherwise, no point is scored. The UOC subscale (34 items) asked participants to judge whether a statement was true (nicotine in cigarettes can be used to relieve depression) and then to judge how confidence when they answered the question (on a scale of 50–100%). Their score was calculated through 1 subtract the absolute difference between correct answers rate and mean of confidence. RP and SN is used to evaluate belief assessment; RF and SC is used to evaluate value assessment; DR is used to evaluate integration; and UOC is used to evaluate meta-cognitive ability. A higher score in each of these sub-scales indicates higher core decision-making skills.

### Need for cognition scale

The NFC scale, developed by Cacioppo and Petty (1984), consists of 18 items. Some examples are “The notion of thinking abstractly is appealing to me”, and “Thinking is not my idea of fun”. In this study, participants were asked to rate the extent to which each of 18 items describes them on a five-point Likert scale ranging from “strongly disagree” to “strongly agree”. The mean value of all items was regarded as the total score of the NFC.

### Statistical analysis

First, Repeated ANOVAs were conducted to compare the fatigue levels, IGT scores, and DMC scores among the nurses between the day shift and the night shift. Then, the difference value of fatigue levels, IGT scores, and A-DMC scores between the day shift and the night shift of participants were calculated (marked as ∆fatigue, ∆IGT, ∆DMC), a rank correlation analysis was conducted to explore the relationship among ∆fatigue, ∆IGT and ∆DMC. Finally, for the purpose of exploring the relationship among NFC, DMC and IGT scores, the participants were divided into high-NFC group and low-NFC group, the independent-samples T test was conducted to compare the difference of DMC and IGT scores between the two groups. And correlation analysis was conducted to ascertain their relationship further. All analyses were conducted on SPSS 20.0 for windows.

## Results

Results showed that there was no significant difference on demographic variables and the number of months the nurses had been working between Group A and Group B (p > 0.05). Meanwhile, no significant differences were found between the two group in IGT performance and DMC scores after both a day shift and a night shift (p > 0.05). Therefore, the data in Group A and Group B was analyzed together.

Table 1 shows the difference in the fatigue levels, IGT scores, and DMC scores among the nurses after the day shift and after the night shift. Repeated measurement by means of the F-test shows that the fatigue levels of nurses after the night shift were significantly higher (F = 188.46, p < 0.01), and the IGT scores (F = 27.77. p < 0.01) and three DMC dimensions, including RF (F = 6.84, p = 0.01), RP (F = 5.84, p = 0.02), DR (F = 5.36, p = 0.02), all significantly decreased, as compared with the participants’ results after the day shift. According to phase division of IGT provided by De Visser et al.^37^, further analysis shows that the decrease in the IGT scores is mainly reflected during the exploitation phase of the testing; namely, during test blocks 2–5 (p < 0.05), and the exploration phase of blocks 1 did not decrease significantly (F = 3.01, p = 0.10), see Table 1.

**Table 1:** Differences in fatigue, IGT, and A-DMC scores in the day and night shifts (n = 107).

	Day shift	Night shift	F
Fatigue	1.79 ± 1.18	4.21 ± 1.36	188.46**
IGT total score	16.06 ± 29.16	− 5.27 ± 31.71	27.77**
Score in IGT block 1	− 3.92 ± 7.54	− 3.23 ± 7.07	3.01
Score in IGT block 2	1.79 ± 7.89	− 1.20 ± 7.17	7.57*
Score in IGT block 3	5.08 ± 8.33	− 0.60 ± 9.90	20.56**
Score in IGT block 4	6.35 ± 9.92	0.19 ± 9.55	23.66**
Score in IGT block 5	4.65 ± 10.62	− 0.58 ± 10.11	16.33**
DR	0.64 ± 0.17	0.59 ± 0.20	5.36*
SC	3.99 ± 1.00	3.92 ± 0.82	0.32
RP	0.71 ± 0.09	0.68 ± 0.10	5.84*
SN	0.37 ± 0.33	0.37 ± 0.31	0.04
DR	0.64 ± 0.17	0.59 ± 0.20	5.36*
UOC	0.86 ± 0.11	0.85 ± 0.10	1.28

*IGT* Iowa Gambling Task, *RF* resistance to framing, *SC* resistance to sunk costs, *RP* consistency in risk perception, *SN* recognizing social norms, *DR* applying decision rules, *UOC* under/overconfidence.

*p < 0.05; **p < 0.01.

The participants’ fatigue levels, IGT scores, and sub-scale scores of A-DMC after the day shift were subtracted by the corresponding scores after the night shift, and the differences (marked as ∆fatigue, ∆IGT score, ∆RF, ∆SN, ∆UOC, ∆DR, ∆RP and ∆SC) were subjected to rank correlation analysis. Results show that ∆fatigue is significantly and negatively correlated with ∆IGT scores, ∆RF, ∆SN, ∆DR which indicated that fatigue caused by nightshift work impaired decision making and DMC in nurses; Moreover, ∆IGT is significantly and positively correlated with both ∆RF and ∆DR; ∆RF positively correlated with ∆SN, ∆DR and ∆SC significantly; ∆SN significantly and positively correlated with ∆DR and ∆SC; ∆UOC positive correlated with ∆SC significantly; however, ∆RP negatively correlated with ∆SC significantly (Table 2).

**Table 2:** Correlation analysis of the changes in fatigue, IGT scores, and A-DMC after night shift (n = 107).

	1	2	3	4	5	6	7
1. ∆Fatigue							
2. ∆IGT score	− 0.26**						
3. ∆RF	− 0.24*	0.36**					
4. ∆SN	− 0.19*	0.02	0.31**				
5. ∆UOC	− 0.01	0.02	0.14	0.14			
6. ∆DR	− 0.31**	0.26**	0.40**	0.22*	0.12		
7. ∆RP	− 0.01	0.05	0.14	− 0.10	− 0.12	0.09	
8. ∆SC	− 0.09	0.13	0.19*	0.22*	0.27*	0.16	− 0.22*

*IGT* Iowa Gambling Task, *RF* resistance to framing, *SC* resistance to sunk costs, *RP* consistency in risk perception, *SN* recognizing social norms, *DR* applying decision rules, *UOC* under/overconfidence.

*p < 0.05; **p < 0.01.

Then, the participants were ranked by scores of NFC. The top 50% were named the high-NFC group, and the remaining 50% were named the low-NFC group. Results show that significant differences between groups may be found in ∆IGT, ∆RF, ∆DR, but not in ∆fatigue; see Table 3. Correlation analysis shows that in the high-NFC group, ∆Fatigue is not significantly related with ∆IGT, ∆ RF, or ∆ DR, but in the low-NFC group, ∆Fatigue is significantly and negatively correlated with ∆IGT, ∆ RF, and ∆ DR; those results indicated that NFC moderates the impairment of decision making, especially in IGT, RF and DR, see Table 4.

**Table 3:** Changes in fatigue, IGT scores, and A-DMC in Low and High NFC groups.

	Low NFC (n = 53)	High NFC (n = 54)	t
∆Fatigue	− 2.68 ± 1.87	− 2.15 ± 1.74	− 1.52
∆IGT score	34.87 ± 45.74	7.74 ± 32.27	3.55**
∆RF	0.36 ± 0.75	0.03 ± 0.73	2.34*
∆SN	0.02 ± 0.44	0 ± 0.40	0.19
∆UOC	0.01 ± 0.14	0.02 ± 0.12	− 0.19
∆DR	0.11 ± 0.23	− 0.01 ± 0.22	2.86*
∆RP	0.05 ± 0.16	0.01 ± 0.11	1.29
∆SC	0.17 ± 0.90	− 0.03 ± 1.48	0.86

*IGT* Iowa Gambling Task, *RF* resistance to framing, *SC* resistance to sunk costs, *RP* consistency in risk perception, *SN* recognizing social norms, *DR* applying decision rules, *UOC* under/overconfidence.

*p < 0.05; **p < 0.01.

**Table 4:** Correlation analysis of the changes in fatigue, IGT scores, and A-DMC in Low and High NFC groups.

		∆IGT score	∆RF	∆SN	∆UOC	∆DR	∆RP
**Low NFC group**							
∆Fatigue	− 0.27^*^	− 0.33^*^	− 0.19	− 0.09	−.28^*^	0.13	− 0.19
∆IGT score		0.30^*^	0.04	− 0.13	0.23^+^	− 0.01	0.16
**High NFC group **							
∆Fatigue	− 0.13	− 0.15	− 0.17	0.03	0.14	− 0.10	− 0.01
∆IGT score		0.35^**^	− 0.08	0.28^*^	0.14	− 0.06	0.11

*IGT* Iowa Gambling Task, *RF* resistance to framing, *SC* resistance to sunk costs, *RP* consistency in risk perception, *SN* recognizing social norms, *DR* applying decision rules, *UOC* under/overconfidence.

*p < 0.05; ^+^p < 0.1.

## Discussion

In this study that targets nurses, the effect of nightshift work on decision-making has been studied in a self pre-and post-control design. Based on previous studies, we propose that the decrease of DMC due to nightshift work is related to the poor performance in the IGT. We also hypothesize that NFC can buffer the effects of nightshift work on decision-making. Our results support that hypothesis to some extent.

### Lowered performance in the IGT and low DMC scores due to nightshift work and fatigue

As in many previous studies, we find that the IGT scores of nurses after nightshift work were significantly lowered^7,38^. Further correlation analysis revealed that fatigue caused by nightshift work impaired risky decision making, which indicated that the increased fatigue was associated with an increasing tendency to make risky decisions. Moreover, three dimensions of the A-DMC also significantly decline after nightshift work, including Resistance to framing, Consistency in risk perception, and Applying decision rules. Further analysis shows that the decrease in the IGT scores is significantly and positively correlated with the reduction of the factors of Resistance to framing and Applying decision rules, indicating that the decrease in the IGT scores after sleep deficiency is related to the decline of DMC.

The interesting results was that the negative correlation relationship between ∆fatigue and ∆IGT. In the analysis of the current study, the values of fatigue of nurse were negative as that was calculated by dayshift subtract nightshift, however, the values of IGT scores were positive as the dayshift minus the nightshift again. Thus, ∆fatigue and ∆IGT negatively correlated in terms of results when calculate the correlation coefficients. However, the real relationship between the changes in fatigue levels and the changes of IGT scores is the greater the change in fatigue, the greater the change in IGT scores of nurses in the current study, which indicated that the tendency to make risky decisions as the increased fatigue because of nightshift. The relationship between mental fatigue and risky decision making had been studied extensively. Researchers found that impaired decision making quality of participants that with 49 to 75 h of sleep deprivation and lead to increased risk-taking behavior on the IGT scores^7,8^, which was consisted with the results of the current study. The cognitive fatigue manipulation experiment also found out that mental fatigue increased risk-seeking behavior and sensitivity to rewards^39^. The IGT is sensitive to the integrity and functioning of the ventromedial prefrontal cortex. Research also supported that IGT scores decline with mental fatigue was coincided with functional neuroimaging study that showed that mental fatigue is associated with reduced cognitive processes mediated by the prefrontal cortex and varied prefrontal activity patterns during executive functional tasks^8,40^. While rudimentary and in need of duplication, these findings suggest that the higher the fatigue level, may be have a greater particularly adverse effect on the functioning of this brain region of cognitive processes. Therefore, it is important to regularly evaluate nurses’ mental fatigue and risk-taking tendencies, and to arrange work schedules accordingly. To ensure the safe operation of medical, it is important to allocate working time and intensity in reasonable manner, and ensure nurses are provided rest time. Everything possible should being done to reduce the occurrence of mental fatigue, and minimize unnecessary medical negligence caused by human behavior.

DMC is a set of core decision-making skills, and individuals with higher DMC can make more rational and correct decisions^10,41^. We must still ask why the decrease in the IGT scores is only significantly and positively correlated with the decline in the factors of Resistance to framing and Applying decision rules. Based on network analysis, Peng et al.^41^ found that the factors of Resistance to framing and Applying decision rules are the core of A-DMC and can maximally reflect decision-making competence. Moreover, Resistance to framing reflects the judgment of values^10^. As reported, sleep deprivation will result in activity abnormality in brain areas related to value assessment (feedback processing), such as the ventromedial prefrontal cortex, the nucleus accumbens, and the insula, and this then affects a person’s feelings about gains and losses^6,15^. Applying decision rules reflects integration, and better integration processes should result in selecting more appropriate decision rules, and in then executing them more accurately and consistently. In other words, integration consists of numerical ability and executive control capability^10^. Reportedly, inadequate sleep will weaken numerical ability, as well as executive control capability^42,43^. The value assessment, numerical, and executive control abilities all significantly affect decision-making^44,45^. We found that the reward sensitivity of the nurse participants was enhanced when they were under a fatigued state due to nightshift work, their loss sensitivity was weakened, and their numerical ability and executive control abilities were diminished, so they could not make rational judgments and instead chose to persistently take risks in the IGT.

### The moderating effect of NFC

We found that NFC can moderate the effects that nightshift work has on the DMC and IGT scores. The decreasing degrees of IGT and DMC scores (impaired Resistance to framing and Applying decision rules) in the high-NFC group were significantly lower than those in the low-NFC group. This finding can be explained by the dual-process model of decision-making. The dual-process model holds that decision-making involves two processing systems: a heuristic system and an analytic system^46^. The former is intuitive, automated, involves rapid parallel processing, and consumes fewer resources, and thus it is significantly affected by emotion. The latter is slow, serial processing based on cognition efforts, and consumes more cognitive resources. Evans^46^ believed that the heuristic system and the analytic system compete for the dominant role of decision-making. Fatigue due to nightshift work lowers the degree of arousal and causes a deficit in cognitive resources^29^. Under this situation, decision-makers may be more likely to be cognitive misers. Killgore et al.^47^ found that the abnormal activation of the default network is related to irrational risk seeking after sleep deprivation, suggesting that cognitive resources cannot be efficiently moved to the brain areas that control the execution of decision-making tasks. In other words, the effects of nightshift work on decision-making can be regarded as the dependence of decision-makers on the heuristic system after a decrease in cognitive resources^47^. NFC reflects the willingness of individuals to devote cognitive resources to thinking. Individuals with high NFC pay more attention to details, and they are more willing to use the analytic system, which can buffer the effects of inadequate cognitive resources on decision-making. Meanwhile, low-NFC individuals are unwilling to devote cognitive resources to thinking, and in a fatigued state, such individuals depend more on the heuristic system. Hence, they more easily run into the framing effect and other cognitive biases, and they make intuitive judgments^27^, which causes them to performed worse in the IGT.

### Limitations

In the current study, we discuss the relationship between the decrease in IGT scores and the decrease of DMC due to sleep deficiency, and we explore whether NFC can buffer the effect of inadequate sleep on decision-making. We obtain some meaningful findings. However, this study has some limitations. First, this study was conducted in a real-life environment—the real working state and conditions of nurses; however, some variables (sleep deprivation) cannot be controlled in the real world in the same way that laboratory research can control them. For instance, we cannot ensure that the nurses rested well and for a sufficient amount of time on the day before their day shifts. Second, this cross-sectional study does not probe into the causality between variables. Third, the task of completing the scales and the IGT was time consuming and sometimes conflicted with the working schedules of the nurses, so we were able to enroll only a small number of participants. The sample size needs to be further expanded and separate the participants in three groups (“high-NFC”, “middle-NFC”, and “low-NFC”) following a Gaussian repartition to further explore the buffering effect in the relationship between fatigue and decision making in future studies, which could even lead to more interesting results and differences. Fourth, future studied should explore the impact of night work on emotions and have a more in-depth perspective on the work to be done to compensate for the cognitive impairment. The current study only studied the cognition factor such as NFC that plays the moderating role, the intermediate variables that may have impact on the relationship between mental fatigue and decision making needs further expansion and consideration. Additionally, some studies have suggested that there is a gender difference in the effect of mental fatigue on decision making^48^. Future studies are encouraged to enroll both male and female participants.

## Data Availability

The datasets used and/or analyzed during the current study are available from the corresponding author on reasonable request.
